# Proteolytic regulation of mitochondrial oxidative phosphorylation components in plants

**DOI:** 10.1042/BST20220195

**Published:** 2022-05-19

**Authors:** Abi S. Ghifari, Monika W. Murcha

**Affiliations:** School of Molecular Sciences, The University of Western Australia, 35 Stirling Highway, Crawley, Perth, WA 6009, Australia

**Keywords:** degradation, mitochondria, oxidative phosphorylation, protease, proteolysis, respiratory system

## Abstract

Mitochondrial function relies on the homeostasis and quality control of their proteome, including components of the oxidative phosphorylation (OXPHOS) pathway that generates energy in form of ATP. OXPHOS subunits are under constant exposure to reactive oxygen species due to their oxidation-reduction activities, which consequently make them prone to oxidative damage, misfolding, and aggregation. As a result, quality control mechanisms through turnover and degradation are required for maintaining mitochondrial activity. Degradation of OXPHOS subunits can be achieved through proteomic turnover or modular degradation. In this review, we present multiple protein degradation pathways in plant mitochondria. Specifically, we focus on the intricate turnover of OXPHOS subunits, prior to protein import via cytosolic proteasomal degradation and post import and assembly via intra-mitochondrial proteolysis involving multiple AAA+ proteases. Together, these proteolytic pathways maintain the activity and homeostasis of OXPHOS components.

## Introduction

**Figure 1.**
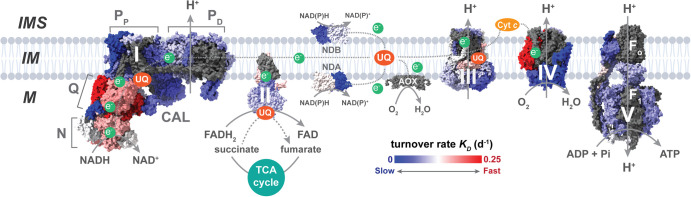
Oxidative phosphorylation (OXPHOS) pathway in plant mitochondria. The electron transfer chain (ETC) consisting of Complex I–IV contribute in generating electron flows (e^−^) necessary for proton (H^+^) pumping from the matrix (M) to the intermembrane space (IMS) across the inner membrane (IM). Complex I modules are indicated: NADH-binding (N), ubiquinone-binding (Q), carbonic anhydrase-like (CAL), proximal proton-pumping (P_P_), and distal proton-pumping (P_D_). Electrons are carried by mobile electron carriers such as ubiquinone (UQ) and cytochrome *c* (Cyt *c*). Plant mitochondria also contain alternative ETC components, such as type II NAD(P)H dehydrogenases NDA and NDB, and alternative oxidase (AOX). Proton gradient is then used by the ATP synthase to drive ATP synthesis. Topographical heatmap depicting turnover rate of individual subunits (per day, d^−1^) in *Arabidopsis thaliana* Columbia-0 ecotype (wild-type) seedlings, from relatively slow (blue) to relatively fast (red). Grey regions represent subunits that were unreliably detected. Data were retrieved from Li et al. [31].

Mitochondria are the central hub for energy production and regulation in most eukaryotes, generating ATP as the energy source for the cell. The majority of ATPs generated by mitochondria are produced by electrochemical machinery located across the inner membrane called oxidative phosphorylation (OXPHOS). OXPHOS utilises proton gradients generated across the inner membrane to produce ATP from ADP and inorganic phosphate. The proton gradient is generated by the electron transfer chain (ETC) consisting of multi-subunit complexes: Complex I (NADH-ubiquinone oxidoreductase); Complex II (succinate-ubiquinone oxidoreductase); Complex III (ubiquinone-cytochrome *c* oxidoreductase); and Complex IV (cytochrome *c* oxidase). Apart from Complex II, which is also involved in the tricarboxylic acid (TCA) cycle, all other ETC complexes pump protons across the inner membrane (IM) from the matrix to the intermembrane space (IMS), forming a supercomplex known as a respirasome [1–3]. Electrons generated from redox reactions by ETC complexes are transferred by mobile electron carriers, such as ubiquinone (UQ) and cytochrome *c* (Cyt *c*). Electrons carried by Cyt *c* are accepted by oxygen at Complex IV, generating water in the process. Proton gradients generated by the translocation of protons powers Complex V (ATP synthase) to synthesise ATP from ADP and inorganic phosphate (Figure 1). In addition to the classical ETC pathway, plant mitochondria also possess alternative electron carrier pathways. Type II NAD(P)H dehydrogenases (NDs) such as NDA, NDB, and NDC can bypass the ETC by generating electrons from NAD(P)H to UQ, while the alternative oxidase (AOX) can transfer electrons from the UQ to oxygen, therefore bypassing Complex III and Complex IV activity (Figure 1).

As an endosymbiotic organelle, the mitochondrion contain its own genome (mitogenome), although it has limited coding capacity and most proteins needed for mitochondrial biogenesis and activity are encoded in the nucleus. In plants, such as the model flowering plant *Arabidopsis thaliana* (Arabidopsis), the mitogenome encodes for *in organello* protein translation machinery such as mitoribosome subunits and tRNAs, and several core OXPHOS subunits [4–6]. Therefore, the majority of OXPHOS components are encoded in the nucleus, translated in the cytosolic ribosome, and imported into mitochondria via various translocation pathways [7]. Consequently, the assembly of OXPHOS complexes requires intricate coordination of protein synthesis, translocation, assembly, and subsequently the maintenance of assembled subunits, a research focus that has emerged in the past 10–15 years. Previous biochemical studies and structural elucidations from various systems demonstrate that OXPHOS complexes assembly is a modular process, involving the stepwise acquisition of subunits to build modules, intermediary complexes, and the holocomplex [8–16].

The modularity of OXPHOS complexes assembly would suggest that a similar stepwise disassembly mechanism exists for quality control. Quality control and degradation of the whole mitoproteome is achieved via mitochondrial autophagy (mitophagy) [17] and to a smaller extent, the formation of mitochondria-derived vesicles (MDV) [18]. Furthermore, there are examples whereby proteins that are imported into mitochondria and misfolded, specifically IM-targeted OXPHOS subunits, can be retro-translocated back to the cytosol to be degraded by ubiquitin-proteasome system [19,20]. In most occasions however, protein homeostasis (proteostasis) and turnover of mitochondrial proteins including OXPHOS components is regulated by intra-mitochondrial proteolysis.

As endosymbiotic organelles, mitochondria contain various internal proteases derived from bacterial ancestors. Most of these proteases belong to the AAA+ (ATPases-Associated with diverse cellular Activity) protein superfamily that is highly conserved across life domains and implicated in functions, ranging from remodelling of cellular membrane and cytoskeleton through protein disassembly [21,22], DNA unwinding [23,24], to proteolysis [25–27] and chaperone [28–30] functions. Recent studies in plants has highlighted the importance of AAA+ proteases in maintaining the mitoproteome, and in particular the OXPHOS complexes. Here, we present recent studies highlighting the proteolytic regulation of OXPHOS complexes in plants, with a focus on the regulation of OXPHOS subunits by proteolytic degradation.

## Regulation of OXPHOS complexes turnover

As the primary contributor to reduction-oxidation (redox) reactions, electron transfer, and the production of reactive oxygen species (ROS), OXPHOS complexes are prone to oxidative damage. This results in a higher turnover rate of OXPHOS subunits compared with the rest of mitoproteome, including proteins involved in protein synthesis and the tricarboxylic acid (TCA) cycle [31]. Furthermore, individual subunits of OXPHOS complexes were observed to exhibit different turnover rates, which suggests a specific degradation mechanism, especially for dysfunctional and damaged subunits.

Individual proteins in the mitoproteome have varied turnover rates depending on their intra-organellar location and function. Even amongst the OXPHOS complexes, turnover rate of individual subunits are highly varied. Protein labelling studies in Arabidopsis indicated that several OXPHOS subunits within the matrix-facing modules of Complex I: NADH-binding (N) and ubiquinone-binding (Q) modules have relatively faster turnover rates, ranging from 1.5–3-fold compared with the average turnover rate of their membrane-bound counterparts (Figure 1) [31,32]. This has similarly been observed in mouse and human cell lines, where Q-module subunits show relatively higher turnover rates compared with the rest of the complex subunits [33]. Interestingly, subunits assembled within intermediates display a higher degree of turnover compared with subunits within the holocomplex [31,34], suggesting a targeted regulation of unassembled subunits.

Regulation of protein turnover can be achieved by different spatiotemporal pathways: (a) pre-import in the cytosol, by the ubiquitin-proteasome system; (b) during import, through retro-translocation or by immediate proteolytic control; and (c) post-import, through the formation of degradation vesicles and whole mitochondrial autophagy (mitophagy), or through proteolysis by intra-mitochondrial proteases.

## Cytosolic degradation of mitochondrial proteins

Nuclear-encoded mitochondrial proteins can be degraded by cytosolic proteasomes prior to import into the organelle. The degradation is mainly following protein import failure, resulting from a decrease in mitochondrial membrane potential, low ATP levels, or the misfolding of mitochondrial precursor proteins [35,36]. Protein import failure can also result from the blockage and disruption of the translocase of outer membrane (TOM) complex, the main protein import pore of the outer membrane [35,37,38]. Failure of protein import into mitochondria can lead to protein accumulation that triggers aggregation in the cytosol [39], therefore degradation of misfolded and aggregated proteins is essential to maintain cellular homeostasis [20,40,41]. However, this mechanism is not restricted to OXPHOS components but applies to most mitochondrial proteins, triggered by protein import failure rather than dysfunctional OXPHOS components.

**Figure 2.**
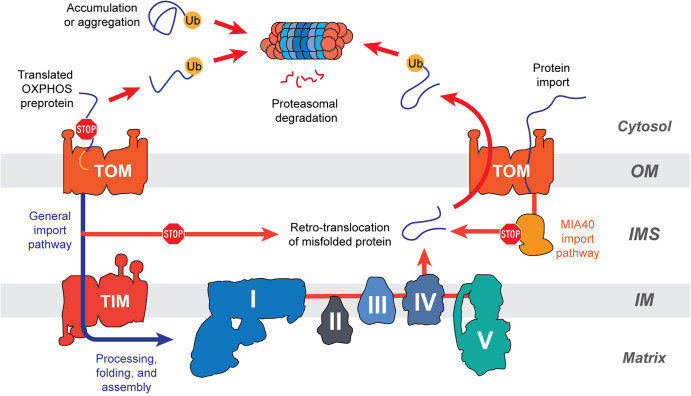
Cytosolic degradation of nuclear-encoded OXPHOS proteins. Failure of OXPHOS components to import is caused by protein accumulation and aggregation in the cytosol or by disruption and blockage of translocase of outer membrane (TOM) transporter which triggers ubiquitination (Ub), leading to proteasomal degradation in the cytosol. Cytosolic degradation may also occur to retro-translocated OXPHOS subunits. Defects in the import and processing of proteins by the intermembrane space MIA40 pathway triggers the retro-translocation of substrates back to the cytosol, rendering the protein prone to ubiquitination and proteasomal degradation.

Retro-translocation of inner membrane imported proteins and degradation by the cytosolic ubiquitin-proteasome system has been observed for uncoupling proteins (UCP) [42] (Figure 2). Studies in murine cells showed that knockout of ubiquitylation and inhibition of cytosolic proteasome resulted in accumulation and slower turnover rate of OM-located UCP2 and UCP3, indicating that these proteins are subjected to proteasomal degradation [42–44]. Further analysis proposed that a linker protein recruits and attaches proteasomes to the membrane surface, which recognises ubiquitylated proteins for degradation [42].

Protein retro-translocation followed by proteasomal degradation has also been observed for proteins translocated via mitochondrial intermembrane space import and assembly (MIA40) pathway [45]. The MIA40 protein import pathway assists the import of IMS-located twin-cysteine containing proteins [7]. In mammals, IM-located OXPHOS subunits have been characterised as MIA40 substrates, including the Complex I subunits NDUFB10 (PDSW) [46], NDUFA8 (PGIV) [47], NDUFS5 (15 kD), and NAD9 [48], in addition to a variety of Complex IV subunits and assembly factors [49–51]. However, since MIA40 import pathway has limited substrates, the cytosolic degradation of OXPHOS subunits may only play a minor role in overall OXPHOS subunit turnover.

## Whole proteomic degradation: mitochondria-derived vesicles and mitophagy

Mitochondria are capable of forming cargo vesicles dedicated to exporting damaged proteins to the peroxisomes and lysosomes (extensively reviewed in [18]). This mechanism is generally used as the last resort in degrading proteins that are unable to be degraded by mitochondrial proteases, such as heavily misfolded and aggregated proteins. In plants, the formation of mitochondria-derived vesicles (MDV) increased following enhanced oxidative stresses, such as prolonged dark photoperiod and senescence [52]. In mammals, MDV has also been implicated in the specific degradation of OXPHOS complex proteins. Inhibition of Complex III with antimycin A (AA) triggered ROS production and the formation of MDV enriched with the Complex III subunit UQCRC2 [53] for degradation by lysosomes [54]. ROS accumulation can also trigger the oxidation and curvature of the mitochondrial membrane cardiolipin, leading to the formation of MDV [18]. MDV is also required to degrade severely aggregated proteins that simply cannot be degraded by mitochondrial AAA+ proteases such as LON and CLPXP [55,56]. This suggests that MDV formation is essential for maintaining quality of mitochondrial proteins in a specific and a smaller scale, particularly under oxidative stress exposure.

In contrast with MDV, mitophagy is a process involving the degradation of whole mitochondria. Mitophagy selectively occurs against severely damaged mitochondria to salvage mitochondrial components in response to severe stress conditions [17]. In plants, mitophagy was widely reported to involve autophagy-related (ATG) proteins, where stress signals received by the Target of Rapamycin (TOR) pathway triggers post-translational modifications (PTMs) such as ubiquitylation, acetylation, and phosphorylation to ATG proteins [57,58]. These PTMs recruit ATG proteins to the damaged mitochondria to form autophagosomes, for transport to the vacuole for degradation [17]. Similar to MDV, mitophagy also appears to be induced with increased oxidative stress in mitochondria. Inhibition of Complex III by AA and myxothiazol or Complex I inhibition by potassium cyanide (KCN) triggered mitophagy [59]. However, in these cases it is likely that mitophagy was triggered by ROS accumulation, rather than in response to OXPHOS inhibition or damaged subunits [17,60].

## Rapid degradation of nascent subunits

Following successful import into mitochondria, cytosolically synthesised OXPHOS precursor proteins undergo additional processing, mainly to remove N-terminal targeting peptide and unstable residues. Targeting peptides are initially cleaved by the mitochondrial processing peptidase (MPP), which in plants, constitutes the cytochrome *bc_1_* complex (Complex III) [61–63]. Additional removal of unstable residue(s) is carried out by the intermediate cleavage peptidase (ICP55) and octapeptidyl peptidase (OCT1) [64–68]. Failure of correct processing results in misfolding and degradation following the N-end rule, whereby certain N-terminal amino acids destabilise the protein and trigger protein degradation [69]. In animals, degradation of misfolded precursors is assisted by the intra-mitochondrial AAA+ proteases such as LONP1 and CLPXP systems [70].

**Figure 3.**
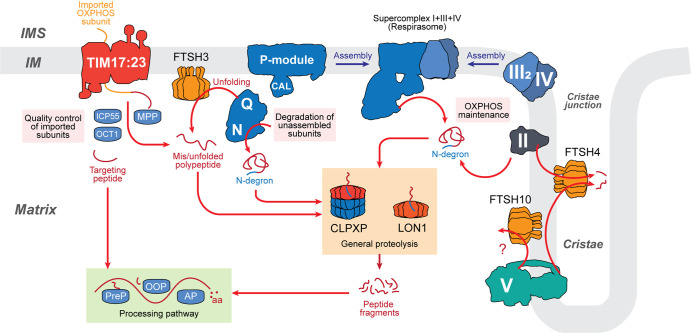
Working model of OXPHOS proteolytic regulation in plant mitochondria. *Quality control of imported subunits.* Nuclear-encoded OXPHOS subunits imported through translocase of inner membrane TIM17:23 complex are subjected to N-terminal processing by mitochondrial processing peptidase (MPP), octapeptidyl peptidase (OCT1), and intermediate cleavage peptidase (ICP55). Improper processing of imported subunits causes misfolding leading to immediate proteolysis. *Degradation of unassembled subunits*. Accumulation of improperly assembled or orphaned OXPHOS modules (for example Complex I NQ-modules) may lead to unfolding by *m*-AAA proteases (FTSH3 and FTSH10) or N-degradation signal (N-degron) tagging followed by proteolysis. Degradation of accumulated subunits is also necessary for proper assembly of OXPHOS complexes. *OXPHOS maintenance*. Damaged OXPHOS subunits can be degraded and replaced in a modular fashion through the disassembly and unfolding by *m*-AAA (FTSH3/FTSH10) or *i*-AAA proteases (FTSH4), followed by N-degron tagging and proteolysis, although the regulation of Complex V by FTSH10 remains unclear. *General proteolysis*. Degradation of unfolded polypeptides, damaged subunits, and N-degron tagged proteins is generally carried out by mitochondrial matrix proteases LON1 and CLPXP. *Processing pathway*. Peptides generated from preprotein processing and general proteolysis are further degraded into amino acids by a series of peptidases, including presequence peptidase (PreP), organellar oligopeptidase (OOP), and aminopeptidases (AP).

Precursor processing generates free peptides that are further degraded by the downstream processing pathways (Figure 3) [71–76]. In this process, peptide fragments are sequentially degraded into smaller fragments by the presequence peptidase (PreP) [77] and organellar oligopeptidase (OOP) [71]. Single amino acids are finally recovered from oligopeptide fragments by multiple residue-specific aminopeptidases (AP) (Figure 3) [73,74,76,78].

## Intra-mitochondrial proteolytic degradation by AAA+ proteases

Plant mitochondria harbour a wide array of both ATP-dependent (AAA+) and ATP-independent proteases that mainly belong to the serine protease family such as degradation of periplasmic protein (DEG) [79] and rhomboid-like proteases (RBL) [80,81]. However, recent studies have highlighted the importance of AAA+ proteases in the disassembly, unfolding, and degradation of OXPHOS subunits. Plant mitochondrial AAA+ proteases are classified into three major classes: AAA or FTSH (Filamentous Temperature Sensitive H) proteases, LON (long filamentous phenotype) protease and CLPXP (caseinolytic protease) machinery. Substrate recognition and specificity depends on their intra-mitochondrial location with IM-bound AAA proteases being either intermembrane space (IMS)-facing (*i*-AAA) or matrix-facing (*m*-AAA). On the other hand, matrix-located proteins are degraded by the soluble matrix-located AAA+ proteases: LON and CLPXP (Figure 3).

**Figure 4.**
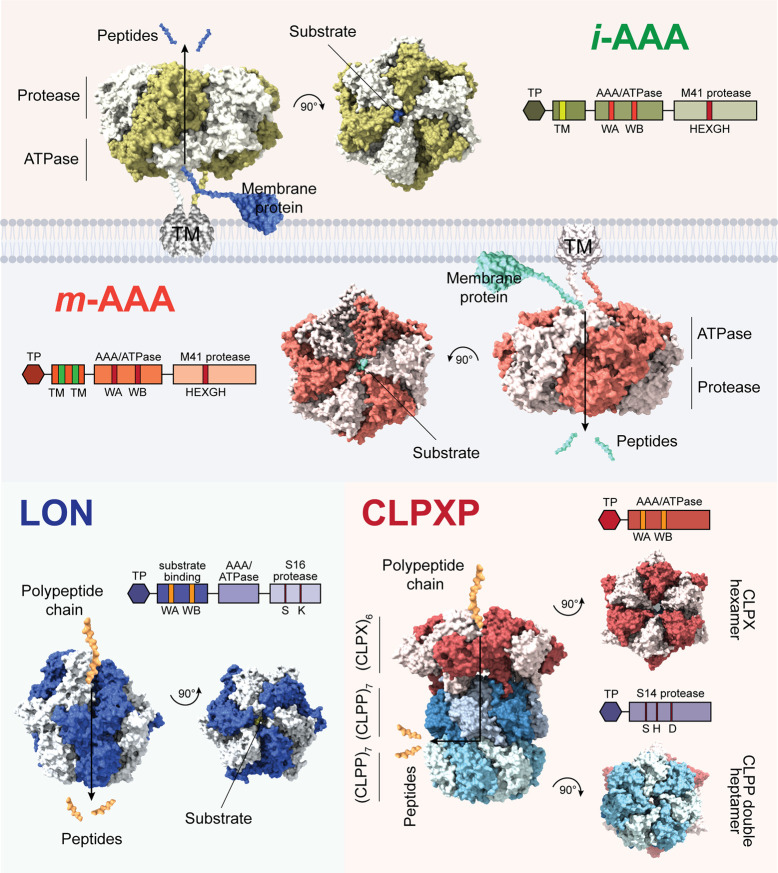
Schematic illustration of the activity and mechanisms of mitochondrial ATPase-associated with diverse activities (AAA+) proteases as inferred from structural studies. *i*-AAA (IMS-facing AAA protease): yeast YME1 (PDB: 6AZ0) [27] homologous to plant FTSH4/11 proteases; *m*-AAA (IMS-facing AAA protease): human AFG3L2 (PDB: 6NYY) [26] homologous to plant FTSH3/10 proteases; LON (long filamentous phenotype protease): *Yersinia pestis* Lon (PDB: 6ON2) [25] homologous to plant LON1; CLPXP (caseinolytic protease system): *Neisseria meningitidis* CLPXP (PDB: 6VFX) [86] homologous to plant CLPXP protease. For each AAA+ proteases, domains are coloured and indicated in linear diagram, with specific features, motifs, and catalytic residues highlighted. Generally, all AAA+ protease sequences contain cleavable targeting peptide (TP) and compose of ATPase that has Walker A and B (WA/WB) nucleotide binding motifs and proteolytic domain. Membrane-embedded *i*-AAA and *m*-AAA contain transmembrane domain (TM) and HEXGH zinc-binding motif within the catalytic site. On the other hand, LON and CLP proteases contain SK dyad and SDH triad as catalytic residues, respectively. Substrate is recognised by ATPase domain and unfolded as the multimeric structure is rearranged upon ATP binding and hydrolysis. The unfolded polypeptide is then translocated into the proteolytic chamber within protease domain for degradation into peptide fragments.

All AAA+ proteases share common structural features and mechanisms that have been reviewed extensively [24,82,83]. Recent cryogenic electron microscopy (cryo-EM) structural determinations of AAA+ proteases further expand our understanding on their mechanistic regulation [25–27,84–86]. AAA+ proteases typically consist of: (1) AAA or ATPase domain that uses ATP to mechanically unfold polypeptide substrates and translocate it to (2) the proteolytic domain, which degrade unfolded polypeptide into smaller peptide fragments (Figure 4). Substrate recognition by the ATPase domain can be mediated by a PTM N-degradation signal (N-degron) tagging and providing an accessible peptide for recognition and binding to start the unfolding process [87,88]. For substrate unfolding, the ATPase domain is mechanically active when forming a multimeric ring (typically a hexamer) with a staircase-like structure (Figure 4). The distinct structural conformation of each ATPase monomer is formed by different binding states with nucleotides (ATP/ADP) mediated by Walker A and B motifs (Figure 4) [25–27,82]. The ATP hydrolysis-driven conformational change of ATPase progressively propels the translocation of polypeptide into the multimeric proteolytic domain, which typically contains either zinc ion (Zn^2+^) cofactor or catalytic serine for peptide degradation [25,84]. In AAA/FTSH and LON proteases, both ATPase and protease domains are encoded by the same gene, which are active as hexamers. In contrast, CLPXP proteolytic machinery consist of hexameric CLPX as ATPase and double-heptameric ring CLPP protease (Figure 4).

## AAA/FTSH proteases

AAA proteases are membrane-bound AAA+ proteases in which both of its ATPase and protease domains are encoded by the same gene and translated into a single polypeptide chain (Figure 4). *i*-AAA proteases typically contain one transmembrane (TM) domain homologous to yeast YME1 and human YME1L [27,75,87], whereas *m*-AAA proteases contain two TM domain homologous to human AFG3L2 [26] (Figure 4). The Arabidopsis genome encodes four mitochondrial AAA proteases: FTSH3 and FTSH10, which are *m*-AAA proteases that can form either homo-hexamer or FTSH3/10 hetero-hexamer [89,90]; and FTSH4 and FTSH11, *i*-AAA proteases found to be exclusively homo-hexameric [69]. Typically, all FTSH proteolytic domains contains a Zn-binding HEXGH conserved domain and are categorised as belonging to the M41 metalloprotease family [27,91].

Recent studies have begun to decipher the intricate role of FTSH proteases play in the proteolytic regulation of OXPHOS complexes and pathway. In Arabidopsis loss of function mutants for either *m*-AAA proteases FTSH3 or FTSH10 had no observable effect to plant growth under both normal laboratory and field conditions [91–94], although the abundance and activity of Complex I and V were reduced, which implicates their role in the assembly and homeostasis of these complexes [92]. Moreover, the effects were even more pronounced in the double knockout of *ftsh3/ftsh10*, as it displayed a decrease in the abundance and activity of Complex I, supercomplex I + III, and Complex V, which consequently resulted in delayed plant growth [94]. However, the mechanistic role of FTSH10 in the regulation of Complex I or V remains unclear and warrants further investigations (Figure 3).

Whilst no direct impact could be observed to plant growth under native physiological conditions, FTSH3 was reported to play a role in the restoration of plant growth in Complex I defective mutants [91]. A forward genetics approach using a knockdown of the *Complex I assembly factor-1* (*ciaf1*) generated a revertant mutant with restored plant growth and Complex I activity [8]. CIAF1 is a LYR domain-containing assembly factor proposed to be involved in iron-sulfur cluster recruitment for Complex I matrix arm domain subunits [8]. The *ciaf1* mutant was previously characterised to have a defective Complex I lacking the matrix arm domain (NQ-module), resulting in reduced overall Complex I activity and subsequently plant growth [8]. The revertant mutant of *ciaf1* contains a point mutation within the FTSH3 ATPase domain, resulting in increased Complex I activity and abundance [91]. Proteomics analyses of this line further revealed that the mutation partially restored the abundance of NQ-module subunits, suggesting the activity of FTSH3 ATPase domain in NQ-module disassembly and overall FTSH3 role in maintaining Complex I homeostasis (Figure 3) [91].

In contrast with FTSH3, a loss-of-function knockout of FTSH4 exhibits a severe growth phenotype when plants are grown under mild heat stress (30°C) conditions, exhibiting increased ROS production, subsequently accumulated carbonylated proteins and decreased ATP synthesis [95]. Furthermore, a decreased abundance of Complex V subunits was observed, indicating the role of FTSH4 in maintaining Complex V stability and activity under stress conditions [95]. FTSH4 has also been reported to be involved in Complex I maintenance under long-term heat stress. During heat stress, mitochondrial proteins are prone to misfold and aggregate, and the lack of FTSH4 reduced the capacity to degrade aggregated proteins [95]. A proteomics study on the FTSH4 knockout indicated that Complex I matrix arm subunits such as Nad9 and PSST, and membrane-bound MNLL and B14.7 were found as aggregates, hinting their possibility as FTSH4 substrates [96]. FTSH4 activity was also reported to be involved in succinate dehydrogenase/SDH (Complex II) regulation based on multiple reaction monitoring (MRM) of heavy nitrogen-labelled (^15^N) peptides [97]. Trapping approach of FTSH4 further affirms its role in Complex II regulation as two of its subunits SDH1 and SDH5 were found to be FTSH4 binding partners [98]. FTSH4 proteolytic activity was also proposed to be required for the degradation of a seed-specific SDH2 paralogue SDH2–3 (Figure 3), whilst its chaperone activity was needed for folding and assembly of the other two paralogues (SDH2–1 and 2) [97].

## LON protease

Similar to FTSH, LON proteases also contain ATPase and proteolytic domains. Cryo-EM structure of the LON protease from bacterium *Yersinia pestis* revealed a spiral staircase-like substrate translocation mechanism conserved in other AAA+ proteases (Figure 4) [25]. LON is a matrix soluble protease belonging to the S16 serine protease family, containing Ser-Lys dyad catalytic residues (Figure 4) [75,99]. In *Escherichia coli*, putative LON substrates have been identified using stable isotope labelling with amino acids in cell culture (SILAC) and protease trapping [100]. The potential substrates identified include enzymes involved in amino acid biosynthesis, sugar and fatty acid metabolism, in addition to the Complex I subunit NuoB [100], a homologue of the Arabidopsis Complex I subunit PSST [16]. In animal systems, a LON-like protease LONP1 has also been identified to play a role in the degradation of inner membrane protein import components and mitogenome regulatory modulators such as mitochondrial transcription factor A (TFAM), and OXPHOS subunits [70,101–103]. Moreover, a recent study in human cell lines highlighted the independent chaperone function of LONP1 to assist in the folding and maturation of a subset of mitochondrial proteins, including OXA1L, an OXPHOS assembly factor, and NDUFA9, a Complex I Q-module subunit [104]. Interestingly, human LONP1 was also observed to influence the availability and function of the CLPXP system, as it was required for the maturation and degradation of CLPX [70], while its knockdown triggered aggregation and reduction in CLPP abundance [105].

In plants, two LON homologues are known to be mitochondrial, LON1 which is exclusively mitochondrial-targeted [99], and LON4 which is dual-targeted to mitochondria and chloroplasts [106]. LON4 has a relatively lower expression compared with LON1, and its function is yet to be determined [107], whereas LON1 has been widely characterised in plants. Loss-of-function *lon1* mutants exhibited delayed plant growth, reduced succinate oxidation and cytochrome *c* oxidation, related to Complex II and IV activity, respectively [99]. Moreover, the activity of several TCA cycle key enzymes were also significantly reduced [99] corresponding to a decrease in the abundance of TCA cycle enzymes [108]. Similar to animal LONP1, plant LON1 acts as general protease that degrades OXPHOS aggregates, while also independently acting as chaperone to assist folding of newly imported proteins [31]. Although further experimental verification is needed, these studies suggest the plant LON1 protease may play a role in general proteolysis of matrix proteins and as a chaperone that maintains the homeostasis of matrix-facing membrane-anchored subunits (Figure 3).

## CLPXP proteolytic machinery

CLPXP is the only multi-heteromeric mitochondrial AAA+ protease that arguably has the most distinctive structural features and activity mechanisms. Consisting of stacks of multimeric complexes, the ATPase and protease domains are encoded by different genes. In plants, CLPXP is composed of a CLPX hexamer as the ATPase, and double-stacked CLPP heptamer (tetradecamer) as the proteolytic domain (Figure 4) [109,110]. Sequential hydrolysis of ATP in CLPX allows the unfolded substrate to be directionally translocated to the symmetric CLPP proteolytic chamber for degradation [84–86]. CLPP was able to form stable heptameric rings without CLPX, although it has a limited and unspecific protease and peptidase activity, with a decreased activity for longer peptides and larger unfolded proteins [111,112]. Although CLPP protease activity does not require ATP hydrolysis, the CLPX ATPase activity is required to enhance substrate access and catalytic efficiency of CLPP by unfolding the protein substrate and translocating it into the proteolytic chamber [111].

The CLPP protease is a member of S14 serine protease family that has a serine-histidine-aspartate triad as its proteolytically-active catalytic residues [75]. Arabidopsis contains three CLPX chaperones: CLPX1–CLPX3, and six CLPP protease core subunits CLPP1–CLPP6, where only CLPP2 is targeted to mitochondria and the remainder are located in the chloroplasts [69,109]. Complete loss-of-function chloroplastic CLPP resulted in embryo lethality and severe developmental phenotypes, indicating the significance in chloroplast biogenesis and functions [113–115]. In contrast, deletion of the mitochondrial CLPP2, has no obvious impact to whole plant phenotype when grown under normal condition [116]. However, steady-state proteomics revealed that soluble matrix located Complex I NQ-module subunits B14, 24 kD, 51 kD, and 75 kD accumulated in *clpp2* knockout [116] highlighting these subunits as possible substrates. Indeed, a BN-PAGE isolation followed by mass spectrometry identification showed that a free N-module intermediate composes of 24 kD and 51 kD subunits were accumulated in *clpp2* knockouts [116]. This finding was reminiscent to *lon1* knockout, where unassembled NQ-module subunits were more slowly degraded, leading to an accumulation [31]. An accumulation of potential substrates could also be observed for other matrix-facing subunits of OXPHOS complexes, including MPP that is a part of Complex III, and ATP-β and ATP-γ that form matrix-facing F_1_ module of Complex V [116] suggesting that CLPXP has a role in protein degradation of membrane-embedded OXPHOS subunits that protrude the matrix, especially unassembled subunits of submodules (Figure 3).

CLPXP system is the most well-studied proteolytic system in terms of structural determination, substrate identification, and characterisation of activity from a variety of organisms including bacteria, fungi, mammals, and plants [117–119]. Furthermore it is located in multiple plant organelles such as mitochondria, chloroplasts, and peroxisomes [120,121]. A tandem affinity purification (TAP) proteomics study in the aging fungus model *Podospora anserina* revealed several TCA cycle and OXPHOS components as its putative substrates, most notably NDUFS1 and NDUFV1 [122]. Similarly, human CLPXP system has also been proposed to directly regulate disassembly and degradation of matrix-facing Complex I N-module subunits NDUFS1, NDUFV1, and NDUFV2 [33], which are orthologous to plant 75 kD, 51 kD, and 24 kD subunits, respectively. Additionally, peripheral Q-module subunits NDUFS2, NDUFS6, NDUFS7, and NDUFS8 (orthologous to plant NAD7, 13 kD, PSST, and 23 kD, respectively) were also identified as putative CLPXP substrates [33,123].

## Concluding remarks

Although OXPHOS components can be proteolytically regulated in various ways, intra-mitochondrial proteolysis by AAA+ proteases play a substantial role in OXPHOS proteostasis. Biochemical, genetic, and structural studies have provided a deeper understanding on the mechanisms of AAA+ proteases in mitochondrial proteostasis. Historically, AAA+ proteases were considered as general proteases with relatively low selectivity and activity against any non-functional proteins. However, recent studies indicate that AAA+ proteases display unique substrate selectivity and function. AAA+ proteases also appear to modularly regulate OXPHOS components, in a similar fashion to their assembly. This way, proteostasis regulation can be energetically efficient, demanding much lower energy than de novo synthesis and assembly of the whole OXPHOS complexes, and allowing for specific regulation and maintenance.

## Perspectives

- Irregularities and disruption of mitochondrial OXPHOS have been known to be detrimental to organisms, such as fatal diseases in human and developmental delay in plants. Although some aspects of plant mitochondrial OXPHOS complexes assembly have been elucidated, the mechanism of their disassembly and degradation remains to be uncovered, which is equally important for their functions.
- While OXPHOS complexes can be regulated extensively through mitochondrial autophagy (mitophagy) and degradation of whole complexes under certain conditions, most OXPHOS components undergo intra-mitochondrial proteolysis that is modular, similar to their assembly process. Moreover, substrate identification of plant AAA+ proteases indicated that these proteases have substrate preference and specific recognition mechanism, rather than act as a general protease.
- A combination of reverse and forward genetic approach and biochemical characterisations are still warranted to elucidate the proteolytic pathway of mitochondrial OXPHOS components. In addition, structural determination and further biochemical analyses to determine activity mechanism can provide details necessary to build the proteolytic pathway model in plant mitochondria.

## Abbreviations

AAA: ATPase-Associated with diverse cellular Activity
AAP: alanyl aminopeptidase
AP: aminopeptidase
BN-PAGE: blue-native polyacrylamide gel electrophoresis
CIAF1: Complex I Assembly Factor-1
CLP: Caseinolytic protease
cryo-EM: cryogenic electron microscopy
DAP: aspartyl aminopeptidase
DIGE: difference gel electrophoresis
ETC: electron transfer chain
FTSH: Filamentous Temperature Sensitive H
HiRIEF: high-resolution isoelectric focusing
IM: inner membrane
IMS: intermembrane space
iTRAQ: isobaric tags for relative and absolute quantification
LAP: leucyl aminopeptidase
LON: Long filamentous phenotype
MDV: mitochondria-derived vesicle
MRM: multiple reaction monitoring
OOP: organellar oligopeptidase
OXPHOS: oxidative phosphorylation
PreP: presequence peptidase
redox: reduction-oxidation
ROS: reactive oxygen species
SDH: succinate dehydrogenase
SILAC: stable isotope labelling with amino acids in cell culture
TCA: tricarboxylic acid
TIM: translocase of the inner membrane
TOM: translocase of the outer membrane
